# Rectal Dose Is the Other Dosimetric Factor in Addition to Small Bowel for Prediction of Acute Diarrhea during Postoperative Whole-Pelvic Intensity-Modulated Radiotherapy in Gynecologic Patients

**DOI:** 10.3390/cancers13030497

**Published:** 2021-01-28

**Authors:** Eng-Yen Huang, Yu-Ming Wang, Shih-Chen Chang, Shu-Yu Liu, Ming-Chung Chou

**Affiliations:** 1 Department of Radiation Oncology, Kaohsiung Chang Gung Memorial Hospital, College of Medicine, Chang Gung University, Kaohsiung 833, Taiwan; hey1200@adm.cgmh.org.tw (E.-Y.H.); scorpion@cgmh.org.tw (Y.-M.W.); kenny25wed@cgmh.org.tw (S.-C.C.); jessica633@cgmh.org.tw (S.-Y.L.); 2 School of Traditional Chinese Medicine, Chang Gung University, Kaohsiung 833, Taiwan; 3 Department of Medical Imaging and Radiological Sciences, Kaohsiung Medical University, Kaohsiung 807, Taiwan; 4 Center for Big Data Research, Kaohsiung Medical University, Kaohsiung 807, Taiwan; 5 Department of Medical Research, Kaohsiung Medical University Hospital, Kaohsiung 807, Taiwan

**Keywords:** rectal dose, small bowel, IMRT, gynecologic malignancies, diarrhea

## Abstract

**Simple Summary:**

Although the small bowel volume effect for acute diarrhea during radiotherapy has been investigated, no study has reported the influence of rectal dose. We analyzed 108 patients undergoing intensity-modulated radiotherapy after hysterectomy. Acute diarrhea was defined as onset during radiotherapy based on Common Terminology Criteria for Adverse Events (CTCAE) version 3. Both small bowel and rectum dosimetric parameters affected Grade 2 to 3 diarrhea. The high-dose volume effects on the small bowel still play an important role in postoperative intensity-modulated radiotherapy. This is the first large cohort study to demonstrate the role of both IMRT dosimetric factors of the rectum and the small bowel in acute diarrhea in gynecological patients with a previous hysterectomy. A small bowel volume of 39.6 Gy < 60 mL and a mean rectal dose of <32.75 Gy are suggested as constraints to treatment planning.

**Abstract:**

We studied the association of rectal dose with acute diarrhea in patients with gynecologic malignancies undergoing whole-pelvic (WP) intensity-modulated radiotherapy (IMRT). From June 2006 to April 2019, 108 patients with previous hysterectomy who underwent WP IMRT were enrolled in this cohort study. WP irradiation of 39.6–45 Gy/22–25 fractions was initially delivered to the patients. Common Terminology Criteria for Adverse Events (CTCAE) version 3 was used to evaluate acute diarrhea during radiotherapy. Small bowel volume at different levels of isodose curves (Vn%) and mean rectal dose (MRD) were measured for statistical analysis. The multivariate analysis showed that the MRD ≥ 32.75 Gy (*p* = 0.005) and small bowel volume of 100% prescribed (V100%) ≥ 60 mL (*p* = 0.008) were independent factors of Grade 2 or higher diarrhea. The cumulative incidence of Grade 2 or higher diarrhea at 39.6 Gy were 70.5%, 42.2%, and 15.0% (*p* < 0.001) in patients with both high (V100% ≥ 60 mL and MRD ≥ 32.75 Gy), either high, and both low volume-dose factors, respectively. Strict constraints for the rectum/small bowel or image-guided radiotherapy to reduce these doses are suggested.

## 1. Introduction

Acute gastrointestinal (GI) toxicities are common side effects during pelvic radiotherapy. Although they are usually transient and reversible, consequential late effects may be troublesome to management in some studies. It is important to reduce the incidence and severity of GI toxicities as much as possible to improve patient quality of life. Symptoms of toxicity are nausea, vomiting, diarrhea, tenesmus, and abdominal cramping. Diarrhea is the most common symptom used for evaluating toxicity and is caused by radiation damage to the bowels, which leads to impairment of water absorption. Radiation-induced inflammation may cause hypermobility and further impairment of bowel function. The small bowel, colon, and rectum are commonly defined as organs at risk (OARs) in radiotherapy, and the excessive irradiation of these OARs could result in GI complications.

The effects of small bowel volume on acute radiation-induced GI toxicities have been well-studied in different diseases treated by pelvic radiotherapy. Although intensity-modulated radiotherapy (IMRT) can reduce GI complications [1,2,3], studies of the small bowel volume effect have seldom been investigated in patients undergoing IMRT. Wang et al. noted the consequential effect of acute diarrhea and late rectal toxicity [4]. The conclusion implies the role of the rectum in acute diarrhea during pelvic irradiation, although this has seldom been investigated. A suitable model for studying the dosimetric effects of the small bowel and rectum is gynecological malignancies because partial volumes of the small bowel and rectum are irradiated. Therefore, the aim of the present study was to address the dosimetric effects of the small bowel and rectum on acute diarrhea in patients with gynecological malignancies who underwent IMRT.

## 2. Materials and Methods

### 2.1. Patients and Radiotherapy

We established a cohort to investigate the correlation between radiation-induced GI toxicities and dosimetry in gynecological malignancies since 2003. From June 2006 to April 2019, 108 patients who received previous hysterectomy for gynecological malignancies and underwent whole-pelvic IMRT were reviewed in the cohort. Before radiotherapy, all patients underwent immobilization in the supine position using a thermoplastic cast and CT-simulation. The patients were encouraged to avoid emptying their bladders before and during the simulations. Rectum emptying was encouraged but not mandatory. Non-contrast CT images with a 5 mm slice thickness were obtained. Contouring of the clinical target volume (CTV) included the vagina, external iliac, internal iliac, and common iliac lymph nodes. Planning target volume (PTV) was an extension of CTV plus 10 mm in all directions based on our setup error data. The bowel loops of the small intestine, bladder, femoral heads, and rectum were contoured for dosimetric calculation using the Pinnacle treatment planning system (ADAC Laboratories, Milpitas, CA, USA). The rectum was delineated from the level of the anus to the sigmoid flexure. Slice by slice, we tracked the colon from the rectosigmoid colon to the descending colon and the ileocecal junction to the ascending colon. The remaining bowel loops we contoured were defined as the small intestine. The separate loops of visible small bowel were delineated from its lowest extent to 2 cm above the CTV. The constraints of V40 Gy were <30% for the small bowel, <60% for the rectum, <70% for the bladder, and <50% for the femoral heads. In general, 7 fields (30°, 80°, 130°, 180°, 230°, 280°, and 330°) of IMRT were arranged. No image guidance during daily irradiation was performed. The step-and-shoot technique was used to perform beam delivery: the whole-pelvic dose ranged from 39.6–45 Gy. Some patients received a low pelvic or local boost after whole-pelvic IMRT. V10% was defined as the small bowel volume covered at 10% of the isodose curve, and V20% to V100% were also recorded for analysis. Therefore, the V10%~V100% were absolute small bowel volumes (mL) at relative doses. If the V100% was 80 mL in a patient undergoing a 39.6 Gy whole-pelvic RT, then the V100% was still 80 mL in the same patient when the prescribed dose of whole-pelvic RT was increased to 45 Gy. In addition to the small bowel, the mean rectal dose (MRD) was recorded for comparison. We used Common Terminology Criteria for Adverse Events (CTCAE) version 3 for diarrhea grading. We evaluated patients weekly and recorded the onset time of any grade of diarrhea in the chart during radiotherapy. Because medication may affect the grading of acute diarrhea, the principle of medical management of acute diarrhea was based on a previous study [5]. 

### 2.2. Statistics

An independent t-test was used to compare patients with different grades of GI toxicity. A receiver operating characteristic (ROC) curve was used to determine the optimal cut-off of dosimetric data for acute diarrhea, and the area under the curve (AUC) was calculated for comparisons. Similar to the survival analysis, we used the Kaplan–Meier method as an actuarial analysis for calculating the cumulative incidence of acute GI toxicity, and the onset dose of toxicity was recorded at each grade. A log-rank test was used to compare the significance between different groups. The end dose in the evaluation of diarrhea grade was 39.6 Gy because not all patients underwent a whole-pelvic dose of 45 Gy. Therefore, we set the whole-pelvic dose of 39.6 Gy as 100% standardized for dosimetry of the small bowel and the rectum. However, in patients without certain grades of diarrhea, the event of diarrhea was censored, and the final dose was 39.6 Gy. For example, the Grade 1 and 2 doses were 18 and 36 Gy, respectively, in one patient. No Grade 3 diarrhea was noted, and the event of Grade 1, 2, or 3 diarrhea was treated as uncensored at 18 Gy, uncensored at 36 Gy, and censored at 39.6 Gy, respectively. Once toxicity-related interruption was noted; the event of diarrhea was uncensored at the event dose. For example, Grade 3 diarrhea was noted in a patient at 28.8 Gy, and treatment interruption was allowed. The event of Grade 3 was treated as uncensored at 28.8 Gy. Therefore, the influence of time courses on the occurrence of acute diarrhea was minimized as well as survival analysis.

A multivariate analysis was performed using the Cox regression model with the forward procedure. Age, body mass index (BMI), and dosimetric data were treated as continuous variables. Concurrent chemoradiotherapy (CCRT), diabetes, and hypertension were treated as a binary variable. After determining the optimal cut-off using the ROC curve, a Cox regression model using categorical variables was used to confirm the dosimetric significance. The result was considered significant if *p* < 0.05.

## 3. Results

The characteristics of 108 patients are shown in Table 1. About two-thirds of the patients were diagnosed with endometrial cancer. The dose per fraction of IMRT was 1.8 Gy in all cases. After a whole-pelvic IMRT of up to 39.6~45 Gy, 91 patients received an additional IMRT boost of 1.8–25.2 Gy to the low pelvic, vagina, or gross tumor site. The cumulative incidence of Grade 2 or greater diarrhea at 39.6 Gy was 48.3%. The cumulative incidence of Grade 3 diarrhea at 39.6 Gy was 18.8%.

### 3.1. Dosimetric Data between Acute Grade 0–1 and Grade 2–3 Diarrhea

We compared V10% to V100% and MRD between patients with Grade 0–1 and Grade 2–3 toxicity. Significant differences were noted between patients with Grade 0–1 and Grade 2–3 toxicity at V80% (*p* = 0.044), V90% (*p* = 0.029), V100% (*p* = 0.020), and MRD (*p* = 0.006) (Table 2). We analyzed V10% to V100% and MRD for the prediction of Grade 2–3 toxicity using ROC curve analysis. Significant roles of V80% (AUC = 0.618), V90% (AUC = 0.629), V100% (AUC = 0.629), and MRD (AUC = 0.627) for the prediction of Grade 2–3 toxicity were also noted (Table 3). Moreover, the optimal cut-off was V100% = 60 mL for small bowel volume (sensitivity 65.4% and specificity 64.3%) and was MRD = 32.75 Gy for the rectum (sensitivity 86.5% and specificity 43.9%) as determined by the ROC curve analysis. Based on these analyses, we selected V100% = 60 mL and MRD = 32.75 Gy as the small bowel and rectum dosimetric factors, respectively.

### 3.2. Cumulative Incidence of Acute Grade 2–3 Diarrhea

**Figure 1:**
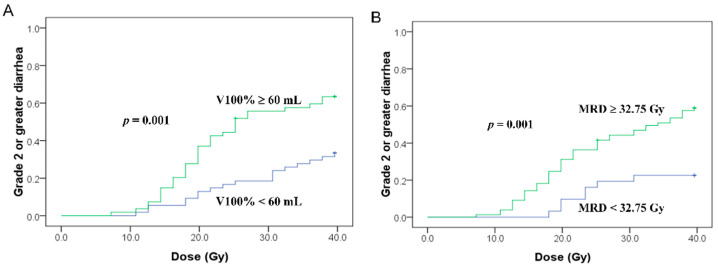
Small bowel volume effect (**A**) and rectal dose effect (**B**) for Grade 2 or greater diarrhea. The horizontal axis is the cumulative prescribed dose to the whole-pelvic target.

The Kaplan–Meier analysis showed that the cumulative incidence of Grade 2–3 diarrhea at 39.6 Gy in patients with <60 mL and ≥60 mL was 33.3% and 63.4% (*p* = 0.001), respectively (Figure 1A). The cumulative incidence of Grade 2–3 diarrhea at 39.6 Gy in patients with MRD < 32.75 Gy and ≥32.75 Gy was 22.6% and 58.8 (*p* = 0.001), respectively (Figure 1B).

### 3.3. Multivariate Analysis of Dosimetric and Non-Dosimetric Data for Acute Grade 2–3 Diarrhea

The multivariate analysis (Table 4) showed that V100% (*p* = 0.005) and MRD (*p* = 0.008) were significant factors for Grade 2–3 diarrhea, while CCRT had a statistical trend (*p* = 0.053). V100% (*p* = 0.006) remained a significant factor for Grade 3 diarrhea.

### 3.4. Combination of Small Bowel and Rectum Dosimetry for Prediction of Diarrhea

**Figure 2:**
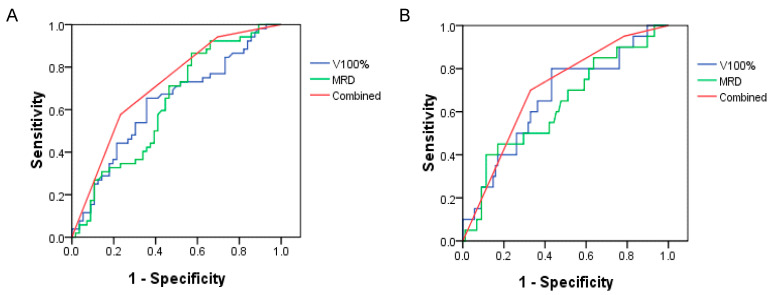
The ROC curve for combination of small bowel volume effect and rectal dose effect on Grade 2 or greater (**A**) and Grade 3 (**B**) diarrhea.

The combination of small bowel and rectum dosimetry revealed a better AUC in the ROC curve analysis (Figure 2). The AUC was 0.627 (*p* = 0.023), 0.629 (*p* = 0.021), and 0.714 (*p* < 0.001) for rectum, small bowel, and combination in Grade 2 or greater diarrhea, respectively. The corresponding AUC was 0.628 (*p* = 0.074), 0.660 (*p* = 0.026), and 0.701 (*p* = 0.005) for Grade 3 diarrhea.

**Figure 3:**
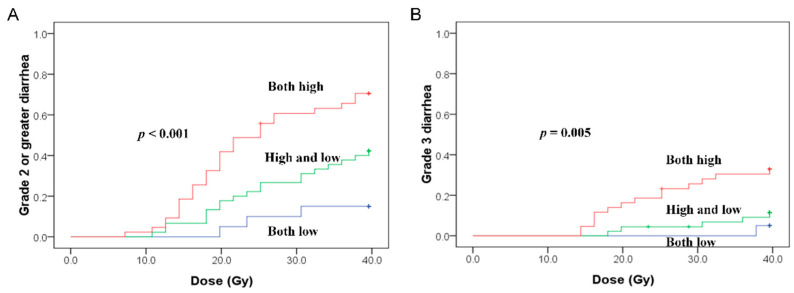
The combination of the small bowel volume effect and rectal dose effect on Grade 2 or greater (**A**) and Grade 3 (**B**) diarrhea. Both high indicates the patient group with V100% ≥ 60 mL and MRD ≥ 32.75 Gy. Either high indicates the patient group with V100% ≥ 60 mL and MRD < 32.75 Gy or V100% < 60 mL and MRD ≥ 32.75 Gy. Both low indicates the patient group with V100% < 60 mL and MRD < 32.75 Gy.

Cumulative rates of Grade 2 or greater diarrhea at 39.6 Gy were 15.0%, 42.2%, and 70.5% in low (V100% < 60 mL and MRD < 32.75 Gy), low/high (V100% < 60 mL and MRD ≥ 32.75 Gy, or V100% ≥ 60 mL and MRD < 32.75 Gy), and both high (V100% ≥ 60 mL and MRD ≥ 32.75 Gy) groups (*p* < 0.001) (Figure 3A), while the corresponding rates of Grade 3 diarrhea were 5.0%, 11.4%, and 32.8% (*p* = 0.005) (Figure 3B).

## 4. Discussion

To date, there are few studies about the effects of small bowel volume in gynecological IMRT patients, and only one study reported rectal dosimetry for acute GI toxicity. Roeske et al. first noted a high-dose (100%) small bowel volume effect in whole-pelvic IMRT patients (*n* = 50), the majority of whom had gynecological malignancies [6], and approximately two-thirds had received hysterectomies. Rectal dosimetry (range 35–49 Gy) was not a significant factor in acute GI toxicity. Isohashi et al. also found a high-dose (90%) small bowel volume effect in whole-pelvic cervical cancer patients (*n* = 62) with radical hysterectomies [7]. Isohashi et al. demonstrated that IMRT (*n* = 30) significantly decreased Grade 2 or higher acute GI complications (63%) in comparison to three-dimensional conformal radiotherapy (3D-CRT) (*n* = 32) (94%) (*p* < 0.01). Furthermore, high-dose (V45 Gy) but not low-dose (V15 Gy) small bowel volumes were significantly smaller in the IMRT group. However, this study did not analyze the small bowel volume effect separately in IMRT and 3D-CRT patients. The IMRT data of acute GI complications (Grade ≥ 2: 63%; Grade 3: 20%) were similar to our data (Grade ≥ 2: 48.1%; Grade 3: 18.5%). Chi et al. found a high-dose (V45 Gy) small bowel volume effect using IMRT in patients (*n* = 32) with endometrial cancer [8]. Furthermore, Li et al. noted that 39% of IMRT patients (*n* = 23) had Grade 2–3 diarrhea, but no significant small bowel volume effects [9]. The literature reviews of acute GI toxicity in patients undergoing IMRT pelvic radiotherapy are listed in Table 5.

The importance of the small bowel in acute GI toxicity could be emphasized through the comparison between whole-pelvic and prostate-only irradiation for prostate cancer [13,14]. Therefore, the higher incidence of acute Grade 2 or greater GI toxicity is considered to be caused by increased irradiation of the small bowel in the whole-pelvic patient group. In terms of excluding confounding factors in rectum irradiation, rectal cancer using preoperative CCRT is considered to be an appropriate disease model since the entire rectum is irradiated. Therefore, the incidence of acute GI toxicity is higher, and Grade 3 toxicity can be easily studied [18]. To date, a small number of studies have examined the small bowel volume effect using IMRT for rectal and anal cancer, in which the majority of patients received preoperative or definitive radiotherapy. Arbea et al. found that V10, V15, and V50 Gy of the small bowel were predictors of acute Grade 3 diarrhea [19], whereas Samuelian et al. noted that IMRT could reduce acute GI toxicity in rectal cancer (*n* = 92) [12]; however, dosimetric analysis was not performed in these studies. Xu et al. noted no small bowel volume effect [20], whereas in a study involving definitive IMRT for anal cancer (*n* = 52), Olsen et al. demonstrated a low-dose (V25–V40 Gy) small bowel volume effect [11]. This finding agrees with the results of Huang et al. [5], which demonstrated a low-dose small bowel volume effect in patients without prior abdominal surgery.

In addition to the small bowel, the importance of the rectum in acute radiation-induced GI toxicity has been proposed in patients with gynecological malignancies, and this was a specific aim of the present investigation. Although there are few studies that have examined the role of the rectum in acute radiation-induced GI toxicity in gynecological patients, radiotherapy of pelvic lesions, with an effort to spare the rectum, is a suitable disease model to support a rectal effect.

The most common disease in which to evaluate the most appropriate rectal dose for acute GI toxicity is prostate cancer, in which only the prostate is irradiated, in order to exclude the small bowel effect. In IMRT cases, 13% of patients had Grade 2 or greater toxicity [13], while 29% of patients without a rectal balloon had Grade 2 or greater toxicity [14]; correspondingly, 6% [15] and 8% [16] of patients with a rectal balloon had Grade 2.

Rectal manipulation has also demonstrated the importance of the rectum in acute radiation-induced GI toxicity. Wu et al. hypothesized that the use of a rectal balloon could reduce acute and chronic toxicity in patients receiving IMRT for postoperative gynecological malignancies [10]. No small bowel dosimetry was analyzed in this study, and the mean percentage of rectal V30 and V40 Gy was 65.1% and 39.3%, respectively. The MRD was expected to be 30–40 Gy, which was similar to the results of our study. Further studies that correlate with rectal dosimetry and acute GI toxicity in patients with prostate cancer can provide stronger evidence for clinical practice. Peeters et al. noted a rectal wall volume effect (mL and % at 30, 35, and 65 Gy) and MRD [21]. Furthermore, a linear trend of 40 Gy rectal volume (%) was demonstrated for acute GI side effects [22]. Dias et al. noted a 25% and 40% rectal volume effect in acute Grade 2 or greater GI toxicity, respectively [23]; Deville et al. demonstrated that infield MRD (Gy), V30 (%), and V40 (%) were important rectal dose parameters [16]. Teh et al. noted that the MRD of Grade 0–1 and Grade 2 were 35.1–35.7 and 38.4 Gy, respectively [15]; this is similar to the difference between Grade 0–1 (33.57 Gy) and Grade 2–3 (35.58 Gy) diarrhea demonstrated in the present study. Based on these studies and the present results, we think that a reduction in rectal dose/volume could result in a decrease in Grade 2 or greater GI toxicity.

The main technique for examining dosimetric effects on acute GI toxicity of the small bowel and rectum is 3D-CRT [18]. In the era of IMRT, a reduction in small bowel and rectum irradiation can be achieved by setting strict dose constraints. Although there have been some clinical data comparison of 3D-CRT and IMRT, no dosimetric data were shown in these studies, and IMRT always limits the small bowel and rectum dose. It is reasonable to hypothesize that IMRT could be used to reduce acute GI toxicity through reduction in the small bowel and rectum dose. In addition, image-guided radiotherapy (IGRT) is a more aggressive technique that can be used to reduce the dose as a result of reducing PTV and OAR exposure of the small bowel [24] and rectum [25].

The current study included a large number of patients undergoing IMRT to study the dosimetric factors of acute radiation-induced GI toxicity (Table 5) in both the small bowel and rectum, which manifests as diarrhea. We used cumulative but not crude incidence for diarrhea, as it is predictable at certain doses during radiotherapy. Therefore, we recorded the onset dose of any grade of diarrhea and the dosimetry correlation to provide adequate information. The distinction between acute and late diarrhea would be helpful in the assessment of other data. Our evaluation time (weekly during RT) was similar to radiation therapy oncology group (RTOG) 1203, the IMRT pelvic radiation for post-operative treatment of endometrial and cervical cancer (TIME-C) trial (weeks 3 and 5) [17].

The limitation of the present study is the inability to perform rectum preparation at each treatment since the medication used for preparation interferes with the scoring of acute GI toxicity. The best model for this application is prostate stereotactic body radiotherapy (SBRT) since the appearance of acute GI toxicity is usually noted after the completion of SBRT (16% of during SBRT to 57% of 1 week from the end of SBRT) [26,27]. Regardless of whether the volume and position of the small bowel and rectum volume vary at each fraction of radiotherapy, the data still show a significant dosimetric correlation to clinical symptoms, in line with the results of a number of related studies. In gynecological patients, the dose of both the small bowel and rectum can be controlled using IMRT and IGRT. The cumulative incidence of Grade 2 or greater diarrhea during whole-pelvic irradiation in the both low-dose (V100% and MRD) group was below 20%. Modern advanced techniques such as proton therapy can be used to spare the GI tract [28] and may further reduce acute GI toxicity; thus, additional techniques warrant study in the future.

## 5. Conclusions

In addition to the small bowel, the rectal dose is the other dosimetric factor involved in acute GI toxicity during whole-pelvic IMRT. Reducing the dose and volume of irradiation of these OARs can decrease the incidence of GI toxicity.

## Figures and Tables

**Table 1:** Characteristics of patients (*n* = 108).

Characteristics	Mean ± SEM or Number (%)
Age (years)	53.6 ± 1.0
BMI (kg/m^2^)	24.7 ± 0.4
Diabetes	
No	96 (88.9%)
Yes	12 (11.1%)
Hypertension	
No	85 (78.7%)
Yes	23 (21.3%)
Disease	
Cervical cancer	32 (29.6%)
Endometrial cancer	72 (66.7%)
Uterine sarcoma	4 (3.7%)
CCRT	
No	84 (77.8%)
Yes	24 (22.2%)
IMRT dose	
WP < 39.6 Gy	5 (4.6%)
WP 39.6 Gy	
No boost	4 (3.7%)
LP 41.4 Gy	1 (0.9%)
LP 45 Gy	43 (39.8%)
LP 50.4 Gy	39 (36.1%)
Local boost to 50.4–64.8 Gy	6 (5.6%)
WP 45 Gy	
No boost	8 (7.4%)
LP 50.4 Gy	1 (0.9%)
Local boost to 64.8 Gy	1 (0.9%)
Diarrhea	
Grade 0	21 (19.4%)
Grade 1	35 (32.4%)
Grade 2	32 (29.6%)
Grade 3	20 (18.5%)
Grade 4	0 (0%)

Abbreviations: BMI = Body mass index; CCRT = Concurrent chemoradiotheray; LP = Low pelvis; SEM = Standard error of mean; WP = Whole pelvis.

**Table 2:** Dosimetric data between Grade 0–1 and Grade 2–3 diarrhea.

Parameter	Grade 0–1	Grade 2–3	*p*-Value
V10% (mL)	434 ± 28	436 ± 26	0.987
V20% (mL)	405 ± 26	415 ± 25	0.763
V30% (mL)	373 ± 24	394 ± 24	0.543
V40% (mL)	328 ± 20	361 ± 22	0.283
V50% (mL)	281 ± 20	311 ± 20	0.286
V60% (mL)	235 ± 17	270 ± 19	0.165
V70% (mL)	192 ± 15	232 ± 17	0.076
V80% (mL)	150 ± 13	189 ± 15	0.044
V90% (mL)	106 ± 10	138 ± 11	0.029
V100% (mL)	61 ± 6	83 ± 7	0.020
MRD (Gy)	33.57 ± 0.58	35.58 ± 0.31	0.006

Abbreviations: MRD = mean rectal dose.

**Table 3:** ROC curve for Grade 2–3 diarrhea.

Parameter	AUC	95% CI	*p*-Value
V10%	0.509 ± 0.056	0.399–0.619	0.873
V20%	0.521 ± 0.056	0.411–0.630	0.712
V30%	0.539 ± 0.056	0.430–0.648	0.483
V40%	0.558 ± 0.056	0.449–0.666	0.303
V50%	0.570 ± 0.055	0.462–0.679	0.210
V60%	0.583 ± 0.055	0.475–0.691	0.136
V70%	0.602 ± 0.055	0.494–0.709	0.068
V80%	0.618 ± 0.055	0.511–0.725	0.035
V90%	0.629 ± 0.054	0.523–0.736	0.020
V100%	0.629 ± 0.054	0.523–0.735	0.021
Rectal dose	0.627 ± 0.054	0.522–0.733	0.023

Abbreviations: AUC = area under the curve; CI = confidence interval.

**Table 4:** Multivariate analyses for Grade 2–3 diarrhea.

Parameter	Grade 2–3			Grade 3		
	HR	95% CI	*p*-Value	HR	95% CI	*p*-Value
Diabetes	−	−	0.884	−	−	0.666
Hypertension	−	−	0.284	−	−	0.724
CCRT	−	−	0.053	−	−	0.106
V100% > 60 mL	2.286	1.282~4.075	0.005	4.622	1.544~1.3833	0.006
MRD > 32.75 Gy	2.980	1.336~6.648	0.008	−	−	0.263

Abbreviations: MRD = mean rectal dose; CCRT = concurrent chemoradiotheray; HR = hazard ratio; CI = confidence interval.

**Table 5:** Literature review of acute GI toxicity in patients undergoing IMRT pelvic radiotherapy.

Author (Reference)	*n*	Disease	OP	Grade 2	Large Fields	Balloon	Small Bowel Volume	Rectum
Roeske et al. [6]	50	GYN	68%	28%	100%	(-)	V45 Gy	No effect
Isohashi et al. [7]	30	cervix	100%	63%	100%	(-)	NA	NA
Chi et al. [8]	32	GYN	100%	34%	100%	(-)	V45 Gy	NA
Li et al. [9]	23	GYN	100%	39%	100%	(-)	No effect	NA
Wu et al. [10]	28	GYN	100%	18%	100%	(+)	NA	NA
Olsen et al. [11]	52	anal	0%	67%	100%	(-)	V25–35 Gy	NA
Samuelian et al. [12]	31	rectal	19%	32%	100%	(-)	NA	NA
Deville et al. [13]	30	prostate	0%	50%	100%	(-)	NA	No effect
Deville et al. [14]	67	prostate	100%	46%	54%	(-)	NA	Dmin
Teh et al. [15]	100	prostate	0%	6%	0%	(+)	NA	No effect
Deville et al. [16]	100	prostate	0%	8%	0%	(+)	NA	MRD
Klopp et al. [17]	122	GYN	100%	26%	100%	(-)	NA	NA
Present study	108	GYN	100%	48%	100%	(-)	V39.6 Gy	MRD

Abbreviations: OP = operation; MRD = mean rectal dose; NA = not applicable.

## Data Availability

The data presented in this study are available on request from the corresponding author. The data are not publicly available due to the nature of this research, participants of this study did not agree for their data to be shared publicly.

## Abbreviations

Radiation therapy (RT); Intensity-modulated radiotherapy (IMRT); Planned target volume (PTV); Clinical target volume (CTV); Gray (Gy); Gynecology (GYN); Common Terminology Criteria for Adverse Events (CTCAE); gastrointestinal (GI); mean rectal dose (MRD); Concurrent chemoradiotheray (CCRT); Whole pelvis (WP); Low pelvis (LP).
